# Association of screen time with self-perceived attention problems and hyperactivity levels in French students: a cross-sectional study

**DOI:** 10.1136/bmjopen-2015-009089

**Published:** 2016-02-26

**Authors:** Ilaria Montagni, Elie Guichard, Tobias Kurth

**Affiliations:** 1 Univ. Bordeaux, UM1219, F-33000 Bordeaux, France; 2 INSERM, HEALTHY Team, UMR1219, F-33000 Bordeaux, France; 3 INSERM CIC1415, CHRU de Tours, Tours, France; 4 Institute of Public Health, Charité – Universitätsmedizin Berlin, Germany

**Keywords:** EPIDEMIOLOGY, Hyperactivity, Attention deficit, Students, Screen time

## Abstract

**Objective:**

To investigate whether high levels of screen time exposure are associated with self-perceived levels of attention problems and hyperactivity in higher education students.

**Design:**

Cross-sectional study among participants of the i-Share cohort.

**Setting:**

French-speaking students of universities and higher education institutions.

**Participants:**

4816 graduate students who were at least 18 years old.

**Exposure:**

Screen time was assessed by self-report of the average time spent on five different screen activities on smartphone, television, computer and tablet and categorised into quartiles.

**Main outcome measure:**

We used the Attention Deficit Hyperactivity Disorder Self-Report Scale (ASRS-v1.1) concerning students’ behaviour over the past 6 months to measure self-perceived levels of attention problems and hyperactivity. Responses were summarised into a global score as well as scores for attention problems and hyperactivity.

**Results:**

The 4816 participants of this study had a mean age of 20.8 years and 75.5% were female. Multivariable ordinary regression models showed significant associations of screen time exposure with quintiles of the total score of self-perceived attention problems and hyperactivity levels as well as the individual domains. Compared to the lowest screen time exposure category, the ORs (95% CI) were 1.58 (1.37 to 1.82) for each increasing level of quintiles of the global score, 1.57 (1.36 to 1.81) for increasing quintiles of attention levels and 1.25 (1.09 to 1.44) for increasing quartiles of hyperactivity.

**Conclusions:**

Results of this large cross-sectional study among French university and higher education students show dose-dependent associations between screen time and self-perceived levels of attention problems and hyperactivity. Further studies are warranted to evaluate whether interventions could positively influence these associations.

**Strengths and limitations of this study**

- This study was conducted in a large, well-defined population of graduate students. To date, epidemiological studies on this specific population are scarce.
- Information was available for television and computer screen time as well as for mobile digital devices.
- Models were adjusted for a priori confounders and intermediate variables of the association between screen time and self-reported attention problems or hyperactivity.
- We did not have information on diagnosed attention deficit hyperactivity disorder (ADHD), but we used a self-reported, self-perceived continuum of attention problems and hyperactivity levels as measured by the validated Adult ADHD Self-Report Scale (ASRS-v1.1). While the full instrument has been found to be meaningful, reliable and valid in French, subscales have not been explicitly tested in this setting.

## Introduction

Young adults, especially students, spend increasingly more time watching a screen on television or on digital devices.^1^ Smartphones, televisions, computers and tablets have become an integral part of young people's lives with higher education students spending most of their screen time surfing the Internet or using a personal computer.^2^

The impact of high screen time on general health and well-being has been investigated previously. Excessive exposure to screen time is associated with unfavourable lifestyle habits and low levels of physical activity,^3^ ^4^ unhealthy eating habits and obesity,^5^ sleep problems^6^ or low vision.^7^

As for mental health, pathologically excessive screen time exposure may lead to substantial consequences. For example, long screen time exposure is a risk marker for anxiety and depression in adolescents and young adults.^8^ Suicide ideation may also be associated with pathologically excessive screen time exposure.^9^ Finally, a high screen time exposure can result in addictive behaviour.^10^

Several studies have reported that excessive exposure to television is associated with attention problems in children of all ages.^11–18^ Despite the increasing prevalence of attention disorder among young adults and the high amount of digital media use among them, data on the potential negative effects of screen time exposure in this group are lacking. Given the available data on adverse effects of screen time exposure, evaluation of its effects among university students is of considerable public health interest. Thus, we aimed to investigate the association of screen time exposure with self-perceived attention problems and hyperactivity levels in a large cohort of French-speaking graduate students per day, excluding holidays. In addition, since the influence of other covariates on this association is less clear, we tested causal association structures which were defined a priori.^19^

## Methods

### Subjects/study population

Study subjects were participants in the ongoing Internet-based Students Health Research Enterprise (i-Share) project, a prospective population-based cohort study of students of French-speaking universities and higher education institutions. The i-Share project was initiated by the Universities of Bordeaux and Versailles Saint-Quentin (France).

To be eligible to participate, a student had to be officially registered at a University or higher education institute, be at least 18 years of age, able to read and understand French and provide informed consent for participation.

Data of this study come mainly from participants from Bordeaux, where active recruitment started in February 2013. Students were informed about the purpose and aims of the study by flyers, information stands at registrations, during lectures, and via social media and newsletters (http://www.i-Share.fr). Furthermore, a group of trained students informed their peers about the study and collected contact information to initiate the online recruitment process. Enrolment followed a two-step process: first, a formal pre-registration on the i-Share online portal was required. In the second step, the student completed the registration process and completed self-administered online questionnaires. Only students who completely filled out the baseline questionnaire were eligible for our analyses. The baseline questionnaire asked information on the participant's health status, personal and family medical history, sociodemographic characteristics and lifestyle habits. We used data available as of 19 March 2015.

### Measures

#### Exposure variable: screen time

Screen time was assessed by self-report of the average time spent on a screen across five different activities: (1) working on a computer/tablet, (2) playing video games on a computer/tablet, (3) surfing the Internet on a computer/tablet, (4) watching television or videos (movies, serials, TV programmes) on a computer/tablet and (5) using a smartphone. Six different time categories could be checked ranging from never to more than 8 h. To summarise the time spent in front of electronic screens, an unweighted scoring system was applied using an arbitrary 6-point scale (never=0, less than 30 min=1, from 30 min to 2 h=2, from 2 to 4 h=3, from 4 to 8 h=4, more than 8 h=5). The score was categorised in quartiles to which were labelled ‘very low’, ‘low’, ‘high’, ‘very high’.

#### Outcomes: self-perceived attention problems and hyperactivity levels

Students were asked to complete questions about their behaviour over the past 6 months based on the 6-item version of the Adult ADHD Self-Report Scale (ASRS-v1.1,^20^ available in various languages at http://www.hcp.med.harvard.edu/ncs/asrs.php). The questions consist of six items providing global information on attention problems and hyperactivity levels. Four items relate to attention problems and two to hyperactivity. We applied the scoring proposed by Kessler *et al*^20^ for each of the six items (never=0, rarely=1, sometimes=2, often=3, very often=4) and summed up the score. Three different scores were calculated: the global score (range from 0 to 24), a score for attention problems (range from 0 to 16), and a score for hyperactivity (range from 0 to 8). The first two scores were categorised in quintiles while the last one was categorised in quartiles. Similar to Kessler *et al*,^20^ we further dichotomised each of the six items (yes/no) and considered participants as having ADHD when they had at least four ‘yes’ responses.

### Statistical analyses

**Figure 1:**
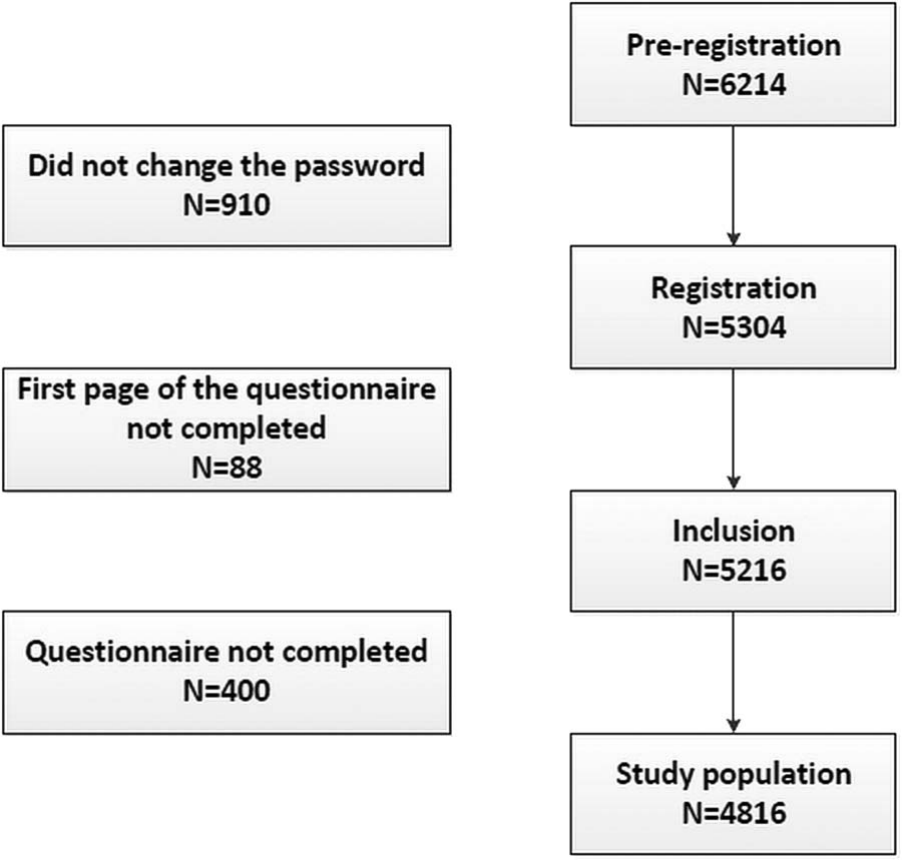
Flow chart of the study population.

Of the 6214 individuals who pre-registered on the i-Share study homepage, 5304 fully registered by changing their password and customising their identification number and 5216 completed the first page of the i-Share questionnaire. For this study, we only included the 4816 participants who fully completed the baseline questionnaire (figure 1).

We compared the characteristics of students with respect to their self-reported screen time measures. We used ordinal logistic regression to calculate ORs and 95% CIs of the association between screen time exposure and self-reported attention problems and hyperactivity. Ordinal logistic regression is an extension of binary logistic regression which allows the outcome variable to have more than two ordered categories. The proportional odds assumption of our ordinal logistic regression was verified and we did not observe any significant violation (p=0.57). Calculated ORs have one reference category for the exposure (in our example, very low screen time) and indicate the difference between increasing outcome categories.

Our main analysis was performed using quintiles of the global score as the dependent variable. We performed additional analyses with self-perceived attention problems and hyperactivity levels as the dependent variables. We also evaluated the association between screen time exposure and ADHD using the definition of ADHD proposed by Kessler *et al*.^20^ ^21^

On the basis of the literature on the magnitude, composition and time distribution of screen exposure, as well as the literature on social and environmental determinants of ADHD,^22–35^ we considered the following covariates for inclusion in our multivariable models: age (18, 19, 20, 21 years or more), gender (male, female), study level (1st, 2nd, 3rd, 4th or higher year of postsecondary education), paid employment while being a student (yes, no), parents’ marital status (divorced, not divorced), parental moral support (absolutely not/a little, moderately, a lot, absolutely yes), self-report of physician-diagnosed depression (yes, no), extracurricular activities (yes, no), sports practice (yes, no), sleep quality (good, quite good, neither good nor bad, bad), recent change in field of study (yes, no), current tobacco consumption (yes, no), alcohol consumption (never, several times per year, once a month, once a week or less, more than twice a week), cannabis consumption (yes, no) and consumption of other drugs (yes, no).

**Figure 2:**
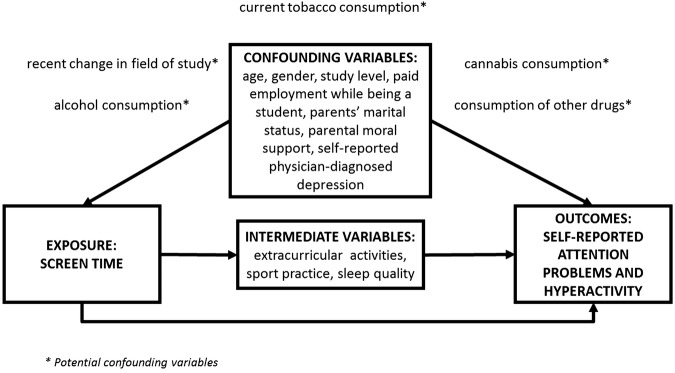
Directed acyclic graph illustrating the variables affecting the association between screen time and self-reported attention problems and hyperactivity.

Following previous studies,^22–35^ selected variables were classified into three groups: confounding variables (ie, variables that are considered causes of both the exposure and the outcome), intermediate variables (ie, variables considered directly affected by exposure and also being a cause of the outcome) and potential confounding variables.^36^ Figure 2 shows the underlying directed acyclic graph, illustrating the three underlying assumptions of our study, and the classification of our adjusted variables in the three aforementioned groups.

In addition to unadjusted analyses, we performed four multivariable analyses: (1) adjusting for the confounding variables, (2) adjusting for the confounding plus the intermediate variables, (3) adjusting for the confounding plus the potential confounding variables and (4) adjusting for all variables. Since there was no meaningful change in the OR between the confounding-adjusted model and the intermediate-adjusted model, we did not further explore potential biases introduced by conditioning on consequences of the exposure in the presence of unmeasured covariates.^19^

In exploratory analyses, we stratified the association between screen time exposure and the global score, attention problem score and hyperactivity score by gender and depression status. We tested for statistically significant effect modification by contrasting the confounder-adjusted model to a model that also included an interaction term between the exposure and either gender or depression status using the likelihood ratio test.

All p values were two-tailed and we considered a p<0.05 to be statistically significant. We performed all analyses using SAS (V.9.3; SAS Institute Inc, Cary, North Carolina, USA).

## Results

The mean age of the participants was (20.8 years, SD 2.8 years) and 75.5% were female. Students reported spending an average of 3.5 h (SD 2.2 h) on a computer/tablet for working, playing or surfing the Internet combined. They also spent on average 4.2 h (SD 3.9 h) watching movies or serials on a TV or computer screen. Finally, they spent 3.1 h (SD 3.6 h) on their smartphone playing games, social networking and surfing on the Internet. Table 1 summarises the personal characteristics of the sample by quartiles of screen time exposure. Participants with very high screen time exposure (ie, highest quartile) were more likely to be older, to have a higher number of years of postsecondary education and to consume higher amounts of cannabis. They were less likely to be female. They were also more likely to be in the highest category of self-reported attention problems and hyperactivity when compared with other participants.

**Table 1:** Characteristics of the study population, i-Share cohort

	Screen time							
	Very low (n=1141)		Low (n=1080)		High (n=1275)		Very high (n=1320)	
	n	Per cent	n	Per cent	n	Per cent	n	Per cent
Quintiles of global score*								
Never	274	24.0	225	20.8	240	18.8	207	15.7
Rarely	190	16.7	194	18.0	221	17.3	200	15.2
Sometimes	221	19.4	238	22.0	248	19.5	258	19.5
Often	293	25.7	258	23.9	333	26.1	381	28.9
Very often	163	14.3	165	15.3	233	18.3	274	20.8
Age (years)								
18	400	35.1	384	35.6	381	29.9	313	23.7
19	228	20.0	192	17.8	244	19.1	207	15.7
20	146	12.8	148	13.7	171	13.4	209	15.8
21 or more	367	32.2	356	33.0	479	37.6	591	44.8
Gender								
Male	244	21.4	249	23.1	298	23.4	387	29.3
Study level								
1st year	582	51.0	553	51.2	565	44.3	479	36.3
2nd year	200	17.5	180	16.7	239	18.7	285	21.6
3rd year	123	10.8	127	11.8	191	15.0	197	14.9
4th year or higher year of postsecondary education	236	20.7	220	20.4	280	22.0	359	27.2
Paid employment while being a student								
No	729	63.9	718	66.5	796	62.4	816	61.8
Parents’ marital status								
Not divorced	795	69.7	750	69.4	868	68.1	890	67.4
Parental moral support								
Absolutely not/a little	136	11.9	110	10.2	117	9.2	138	10.5
Moderately	212	18.6	202	18.7	257	20.2	271	20.5
A lot	462	40.5	445	41.2	501	39.3	504	38.2
Absolutely yes	331	29.0	323	29.9	400	31.4	407	30.8
Self-report of physician-diagnosed depression								
No	1003	87.9	953	88.2	1110	87.1	1138	86.2
Extracurricular activities								
No	802	70.3	753	69.7	851	66.7	891	67.5
Sport practice								
No	546	47.9	531	49.2	628	49.3	674	51.1
Sleep quality								
Good	226	19.8	197	18.2	209	16.4	201	15.2
Quite good	402	35.2	404	37.4	477	37.4	482	36.5
Neither good nor bad	298	26.1	248	23.0	293	23.0	325	24.6
Bad	215	18.8	231	21.4	296	23.2	312	23.6
Recent change in field of study								
No	866	75.9	848	78.5	960	75.3	954	72.3
Current tobacco consumption								
No	783	68.6	708	65.6	840	65.9	844	63.9
Alcohol consumption								
Never	124	10.9	115	10.6	107	8.4	102	7.7
Several times per year	251	22.0	235	21.8	245	19.2	236	17.9
Once a month	185	16.2	184	17.0	225	17.6	226	17.1
Once a week or less	344	30.1	364	33.7	436	34.2	443	33.6
More than twice a week	237	20.8	182	16.9	262	20.5	313	23.7
Cannabis consumption								
No	549	48.1	471	43.6	562	44.1	527	39.9
Consumption of other drugs								
No	947	83.0	913	84.5	1032	80.9	1047	79.3

Numbers may not add to 100% due to rounding values.

*ADHD score categorised in quintiles according to Kessler *et al*.^20^

ADHD, Attention Deficit Hyperactivity Disorder.

Table 2 summarises the association between screen time exposure and three different outcomes, that is, the quintiles of the global score (self-perceived attention problems and hyperactivity levels), the quintiles of the self-perceived attention problems score and the quartiles of the self-perceived hyperactivity levels score based on the ASRS-v1.1 scale. The multivariable ordinal logistic regression showed that increasing levels of screen time were associated with a higher risk of self-perceived attention problems and a higher risk of hyperactivity.

**Table 2:** Association between screen time and self-reported attention problems and hyperactivity

	Quintiles of global score		Quintiles of the score of the self-perceived attention deficit		Quartiles of the score of hyperactivity	
	OR (95% CI)	p Value	OR (95% CI)	p Value	OR (95% CI)	p Value
Univariate		<0.0001		<0.0001		0.03
Very low	1.00		1.00		1.00	
Low	1.07 (0.92 to 1.24)		1.09 (0.94 to 1.26)		1.02 (0.88 to 1.19)	
High	1.26 (1.10 to 1.45)		1.25 (1.09 to 1.44)		1.16 (1.00 to 1.33)	
Very high	1.55 (1.35 to 1.79)		1.58 (1.37 to 1.82)		1.19 (1.04 to 1.37)	
Confounding		<0.0001		<0.0001		<0.01
Very low	1.00		1.00		1.00	
Low	1.08 (1.04 to 1.26)		1.11 (0.95 to 1.28)		1.03 (0.89 to 1.20)	
High	1.28 (1.12 to 1.48)		1.27 (1.10 to 1.46)		1.18 (1.02 to 1.35)	
Very high	1.58 (1.37 to 1.82)		1.57 (1.36 to 1.81)		1.25 (1.09 to 1.44)	
Confounding and intermediate		<0.0001		<0.0001		0.01
Very low	1.00		1.00		1.00	
Low	1.07 (0.93 to 1.25)		1.10 (0.95 to 1.27)		1.02 (0.88 to 1.18)	
High	1.25 (1.09 to 1.44)		1.24 (1.07 to 1.43)		1.16 (1.00 to 1.33)	
Very high	1.54 (1.33 to 1.77)		1.54 (1.33 to 1.77)		1.23 (1.07 to 1.42)	
Confounding and potential confounding		<0.0001		<0.0001		0.01
Very low	1.00		1.00		1.00	
Low	1.09 (0.94 to 1.26)		1.12 (0.96 to 1.30)		1.02 (0.88 to 1.19)	
High	1.29 (1.12 to 1.48)		1.27 (1.10 to 1.46)		1.17 (1.02 to 1.35)	
Very high	1.57 (1.36 to 1.81)		1.56 (1.35 to 1.80)		1.24 (1.07 to 1.43)	
All		<0.0001		<0.0001		0.01
Very low	1.00		1.00		1.00	
Low	1.08 (0.93 to 1.25)		1.11 (0.96 to 1.29)		1.01 (0.87 to 1.17)	
High	1.25 (1.09 to 1.45)		1.24 (1.07 to 1.43)		1.15 (0.99 to 1.33)	
Very high	1.52 (1.32 to 1.76)		1.52 (1.32 to 1.76)		1.22 (1.06 to 1.41)	

Results for ordinal logistic regression models with self-perceived attention problems and hyperactivity levels as the dependent variables and screen time levels as the independent variable. The seven models shown are declined according to the items for ADHD, Inattention levels and Hyperactivity levels. The reference screen time group is the ‘very low’ modality and the reference dependent variable is the ‘never’ modality. Adjusted for confounding set (age, gender, study level, paid activity during studying, parental condition, parental moral support and depression), intermediate set (extracurricular activities, sport practice and sleep quality) and potential confounding set (field of studying changing, current tobacco consumption, alcohol consumption, cannabis consumption and other drug consumption).

Since the results of our four multivariable modelling approaches were similar, we chose to present here results for the model adjusting for potential confounding factors. With regard to the global score, the ORs steadily increased with increasing levels of screen time exposure categories. High screen time exposure was associated with increasing global score quintiles (OR 1.28 95% CI 1.12 to 1.48), which further increased for the very high screen time category (OR 1.58, 95% CI 1.37 to 1.82).

The pattern of the association between screen time exposure categories and self-perceived attention problems was similar to that seen for the global score, but the effect sizes were slightly lower. High screen time and very high screen time were significantly associated with higher levels of attention problems. For the hyperactivity levels, high screen time (OR 1.18, 95% CI 1.02 to 1.35) and very high screen time (OR 1.25, 95% CI 1.09 to 1.44) were significantly associated with higher quartiles of the hyperactivity score.

We also performed secondary analyses using the dichotomous classification of ADHD as our outcome. The results of these analyses were similar to what we observed for the associations between screen time and self-perceived attention problems and hyperactivity. The OR for ADHD was 1.43 (95% CI 1.19 to 1.73) when we compared the highest quartile of screen time to the lowest. Again, results remained essentially unchanged across the different multivariable models.

Finally, there was no indication that the association between screen time and the global score, attention problems score and hyperactivity score was modified by gender and depression status.

## Discussion

In this large cross-sectional study among French-speaking postsecondary students, we found that increasing levels of screen time exposure were associated with increased risk of self-perceived attention problems and hyperactivity levels. Adjustments for covariates that could potentially influence these associations either as confounding variables or as intermediate variables did not result in a meaningful attenuation of the ORs. The association appears stronger for the self-perceived attention problems domain in comparison to the hyperactivity domain.

As highlighted by our data, university students are high consumers of electronic devices spending at least an average of 3 h/day on at least one digital device. They use them for recreational activities, like watching videos or playing online games, and for their work or studies. More and more frequently, computers, smartphones and tablets are used during university courses for taking notes, performing research or for other concentration-demanding tasks.^37^ Understanding how screen time can influence attention problems and hyperactivity levels during this particular period of life is therefore of importance, especially in the light of the increase of ADHD diagnosis on college campuses over the past decades.^38^ ^39^

### Comparison to previous research

Our results are in line with previous studies,^40^ which only assessed screen time exposure from television and video games. We included time spent on portable electronic devices and new technology tools like smartphones and tablets in our definition of screen time, and evaluated the influence of the time spent on these devices on self-reported ADHD features. In contrast with the work of other groups focusing on children^41^ or adolescents,^42^ we employed a large cohort of graduate students. Our study expands on the work of previous studies by demonstrating that among young adults, higher amount of screen time exposure, as measured by time on various devices, is associated with a higher risk of reporting attention problems and hyperactivity. As for gender, previous studies^43^ ^44^ indicated that screen time and prevalence of ADHD were higher in boys and male adolescents. We did not find effect modification by gender, but our population was older (mean age 20.8 years) than in previous studies.

In previous studies, several factors were associated with either screen time or ADHD levels. Age and gender,^24^ paid employment,^29^ parenting style,^25^ sport practice,^32^ sleep quality,^23^ tobacco, alcohol, cannabis and drugs consumption^31^ were associated with screen time exposure. Similarly, age,^30^ gender,^27^ paid activity,^33^ parental situation,^35^ sport practice,^28^ sleep quality,^26^ tobacco and alcohol consumption,^22^ and cannabis consumption^34^ were associated with ADHD. However, very few studies have explored the influence of these factors on the association between screen time and ADHD. One study did examine the association of alcohol and cannabis with both screen time and attention problems and hyperactivity.^45^

### Modelling considerations and potential biological mechanisms

Since screen time has only recently been recognised as an important factor for general health and well-being, the structure of the causal association of how covariates may relate to screen time is not always clear. Thus, we evaluated different model structures to better understand the role of such factors (ie, confounding or intermediate variables).

Since the effect estimates of the various model structures did not show meaningful differences, we conclude that the association between screen time exposure and our outcomes was robust and not influenced by the underlying causal relationship structure of the covariates.

There are several potential biological links that could explain the observed association between electronic screen exposure and ADHD, mainly via cortical network activation^45^ or central visual processing.^46^ ^47^ However, since our data cannot directly test any biological mechanism, we believe it is beyond the scope of this paper to discuss potential mechanisms in detail.

### Strengths and limitations

The strengths of our study include the large number of participants, the standardised assessment tools and available detailed information on potential confounding and intermediate factors. In addition, we focused on an age group that is of particular interest (ie, young adults, particularly those enrolled in universities and higher educational institutions) as this group has high exposure to electronic screens.

Several limitations have to be considered when evaluating our results. First, our study was cross-sectional and we cannot strictly separate the timing of exposure, outcome and the covariates. We conceptualised that high screen time exposure leads to self-reporting of inattention and hyperactivity in college students, but the inverse may also be true. For example individuals with ADHD may isolate themselves more readily than individuals without ADHD and utilise electronic devices more as a consequence of this isolation.^48^ However, this seems to be a less likely scenario than our proposed pathway of high screen time leading to inattention and hyperactivity. Second, we relied entirely on self-reported information and misclassification of collected information is possible. However, we have no reason to believe that misclassification is directly linked to screen time or the outcome events, thus resulting in random misclassification. While the ASRS V.1.1. has been previously used in a population-based setting in France^49^ ^50^ and the total full ASRS-ADHD score has been found to be meaningful, reliable and valid in French adults in a factorial validity study,^51^ subscales have, to the best of our knowledge, not been validated in France. Third, our study reported screen time per device but did not take into account the possibility of contemporary multiscreen viewing, that is, the fact that students can use different digital devices at the same time. We summed up screen time per device, thus potentially overestimating the measure of screen time exposure. Furthermore, we did not ask a specific question assessing the amount of time spent playing video games on a television screen. Fourth, while we had available information on many proposed confounding factors, residual or unmeasured confounding is possible as our study is observational. Lastly, our sample is restricted to students who voluntarily participated in the i-Share project and extrapolation to other populations may be limited. For example, the participants in i-Share are mainly women (about 75%) and are interested in health issues, which may represent a sampling bias. However, we have no reason to believe that the association between screen time exposure and our outcomes would be different in other student populations.

### Potential implications, next steps

Our results indicate that high exposure to electronic screens is associated with self-perceived attention and hyperactivity problems. Since these may affect academic performance and overall well-being, future studies should evaluate whether reducing screen time exposure results in reduction of self-perceived attention and hyperactivity problems in young adults.
